# Supplementary figures and images for: Purkinje cell–specific deficiency in SEL1L-HRD1 endoplasmic reticulum–associated degradation causes progressive cerebellar ataxia in mice
Source: JCI Insight. 2024 Nov 8;9(21):e174725. doi: 10.1172/jci.insight.174725 (PMC11563667; doi:10.1172/jci.insight.174725)

Figure 1G

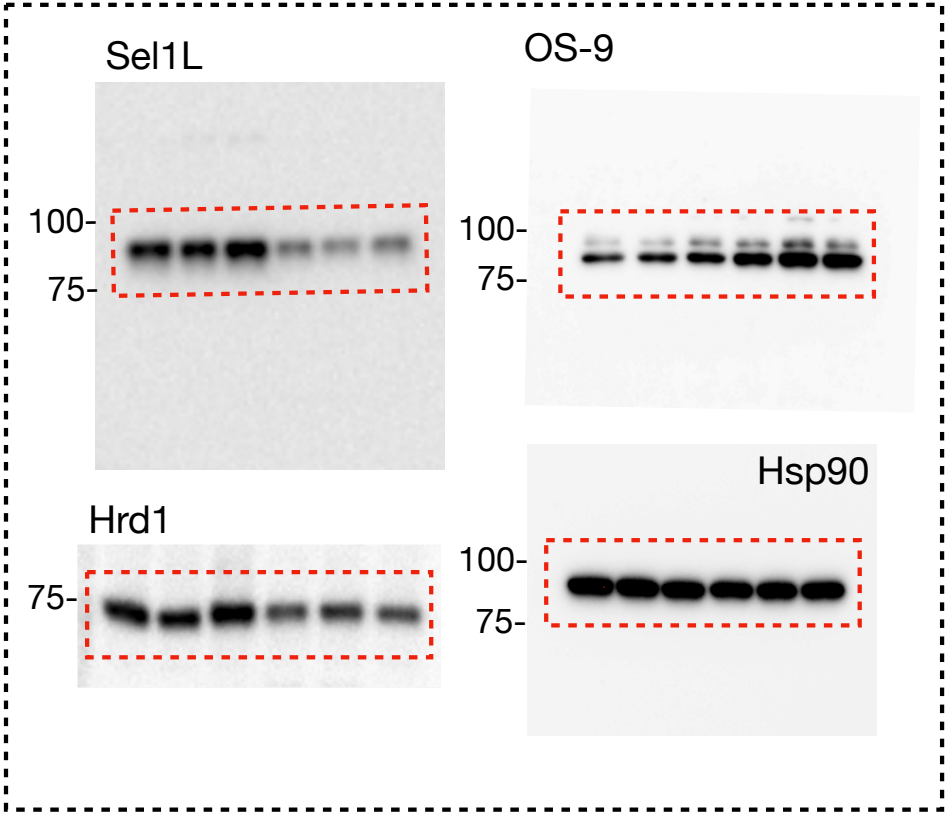

**Figure 4E**

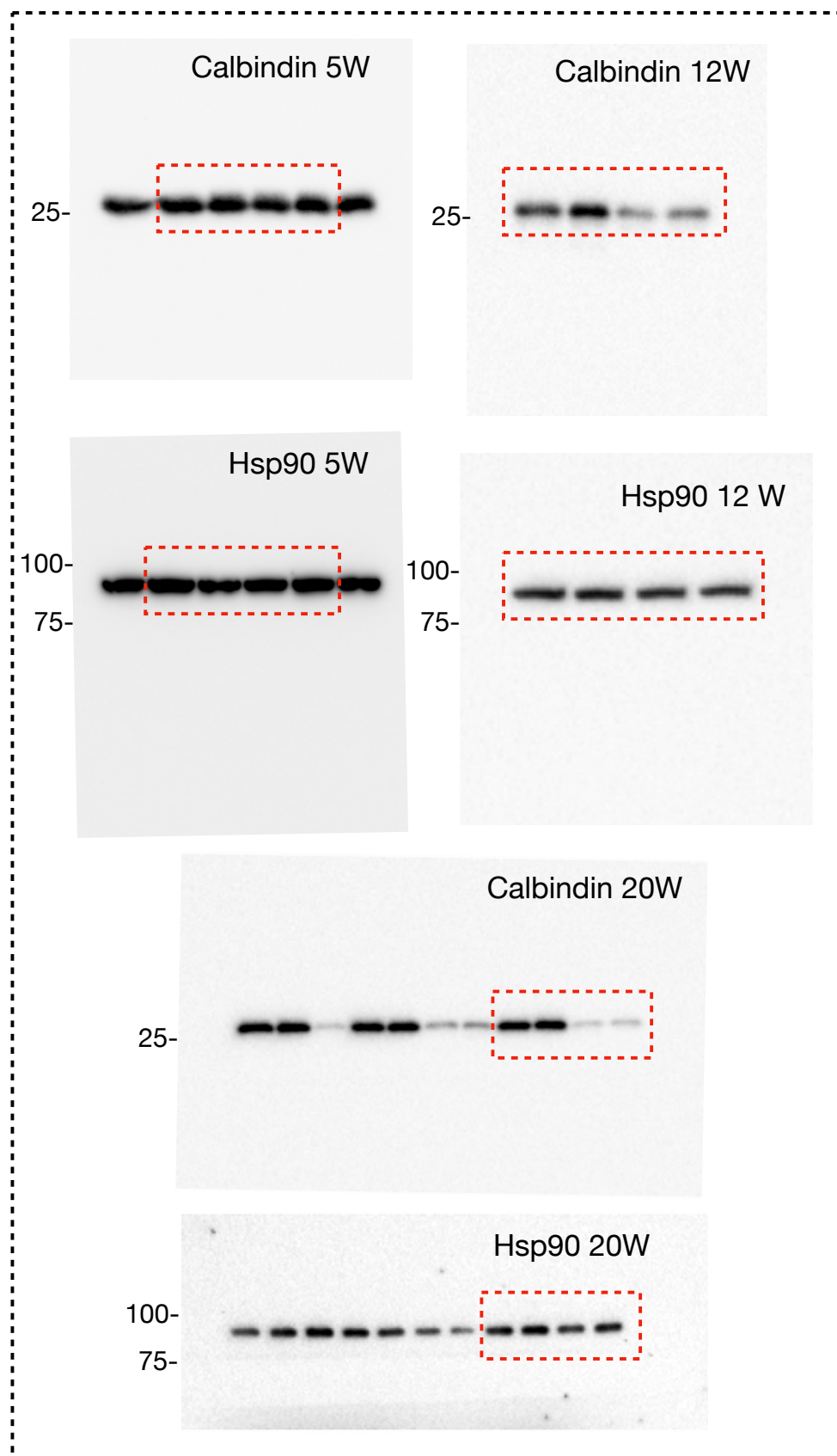

**Figure 6B**

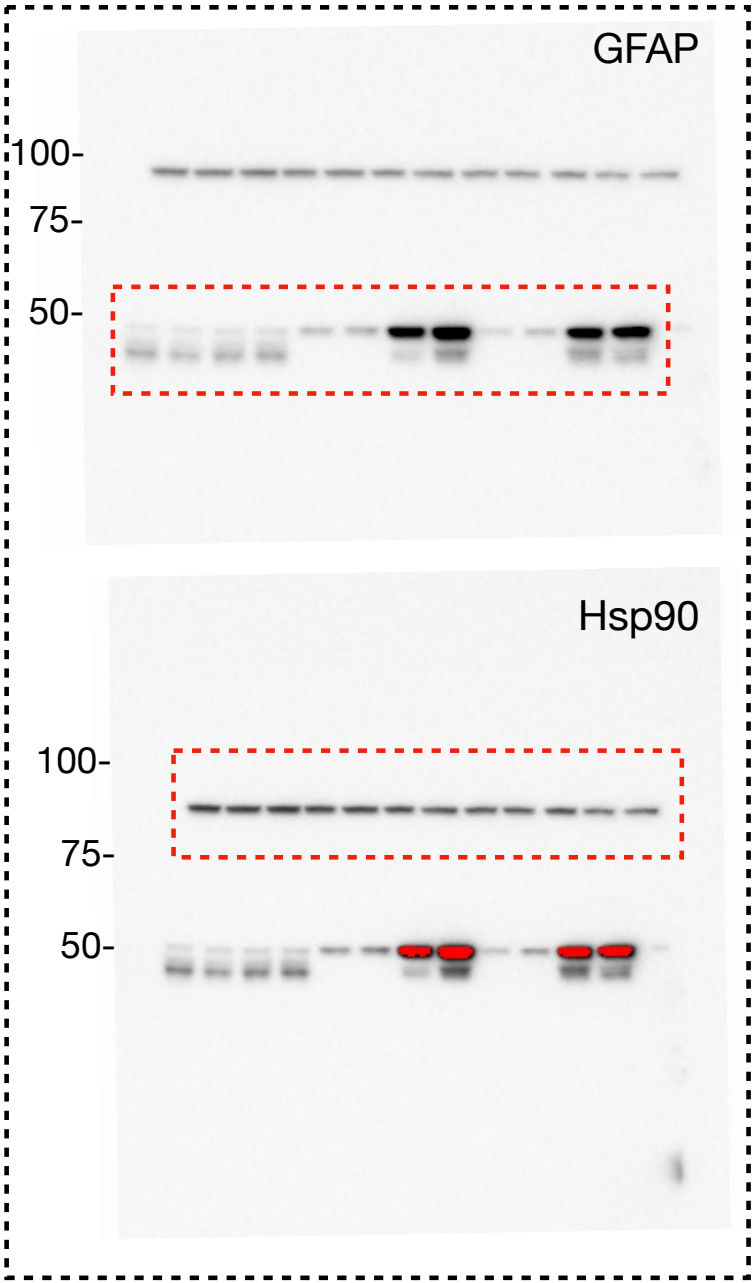

**Figure 7A**

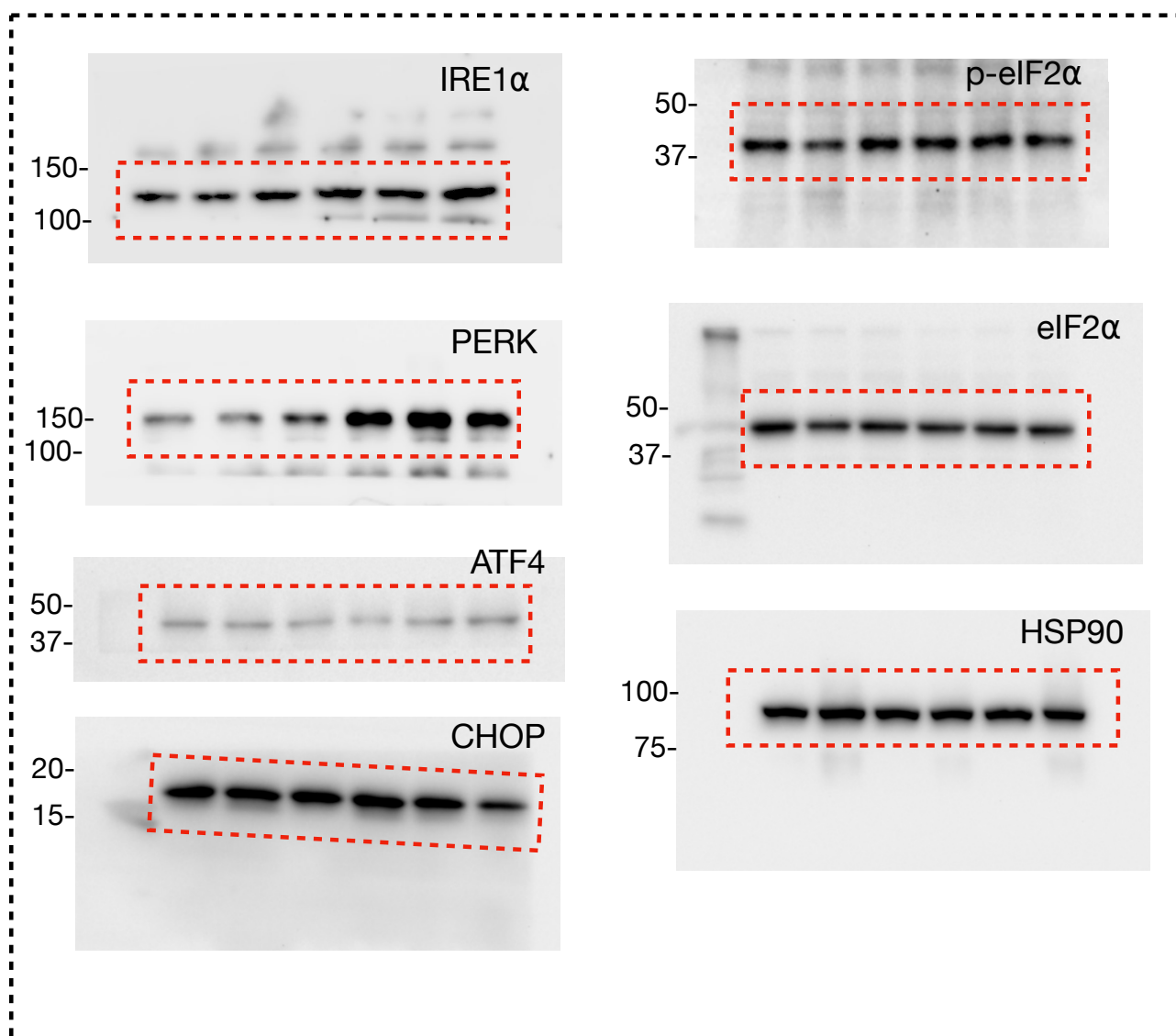

**Figure 7E**

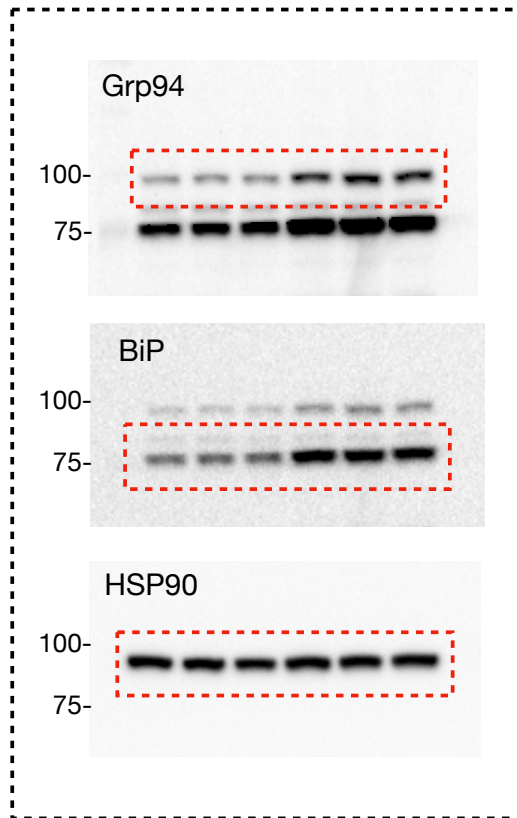

Supplement: Unedited blot and gel images [file jciinsight-9-174725-s329.pdf]
